# Association of lung function with the risk of cardiovascular diseases and all-cause mortality in patients with diabetes: Results from NHANES III 1988-1994

**DOI:** 10.3389/fcvm.2022.976817

**Published:** 2022-09-09

**Authors:** Nian Huang, Chengyao Tang, Shiyang Li, Wenzhi Ma, Xiaobing Zhai, Keyang Liu, Haytham A. Sheerah, Jinhong Cao

**Affiliations:** ^1^Department of Epidemiology and Biostatistics, School of Public Health, Wuhan University, Wuhan, China; ^2^Division of Biomedical Statistics, Department of Integrated Medicine, Osaka University Graduate School of Medicine, Suita, Osaka, Japan; ^3^Department of Epidemiology and Biostatistics, School of Medicine, Wuhan University of Science and Technology, Wuhan, China; ^4^Public Health, Department of Social Medicine, Osaka University Graduate School of Medicine, Osaka, Japan; ^5^Health Promotion and Health Education Research Chair, King Saud University, Riyadh, Saudi Arabia; ^6^Ministry of Health, International Health Regulations, Riyadh, Saudi Arabia

**Keywords:** forced expiratory volume in 1 s (FEV1), forced vital capacity (FVC), cardiovascular disease (CVD), coronary heart disease (CHD), diabetes, National Health and Nutrition Examination Survey (NHANES)

## Abstract

**Objective:**

The potential effects of pulmonary dysfunction on cardiovascular diseases (CVD) and all-cause mortality are receiving attention. The current study aimed to explore whether reduced lung function predicts CVD and all-cause mortality in people with diabetes.

**Methods:**

A total of 1,723 adults with diabetes (mean age 60.2 years) were included in the National Health and Nutrition Examination Survey (NHANES III). Death outcomes were ascertained by linkage to the database records through 31 December 2015. Cox proportional hazards regression models were used to estimate hazard ratios (HRs) and 95% confidence intervals (CIs) for coronary heart disease (CHD), CVD, and all-cause mortalities. We conducted stratified analyses based on age, body mass index (BMI), history of hypertension, and dyslipidemia.

**Results:**

During a mean follow-up of 14.62 years (25,184 person-year), a total of 1,221 deaths were documented, of which 327 were CHD, 406 were CVD, and 197 were cancer. After multi-factor adjustment, participants with lower FEV1 and FVC had a higher risk of CHD, CVD, and all-cause mortality. This association was also found in lower FVC and a higher risk of cancer mortality [HR: 3.85 (1.31–11.32); P for trend = 0.040], but the association of FEV1 was attenuated after adjustment for covariates [HR:2.23 (0.54–9.17); P for trend = 0.247]. In subgroup analysis, we found that the adverse associations of FEV1 and FVC with CVD mortality were observed in subgroups of age, BMI, and history of hypertension and dyslipidemia.

**Conclusion:**

Declined lung function was associated with a higher risk of CVD and all-cause mortality in people with diabetes. Lung function tests, especially FEV1 and FVC, should be encouraged to provide prognostic and predictive information for the management of CVD and all-cause mortality in patients with diabetes.

## Introduction

Several studies have extensively recognized that lung function was associated with the risk of all-cause and cardiovascular risk (1–6). In the general population, lung function, as indicated by a low forced expiratory volume in 1 s (FEV1) and forced vital capacity (FVC), demonstrated an inverse association with coronary heart disease (CHD), stroke, and other cardiovascular diseases mortality (CVD) (7, 8). Several mechanisms have been established for the association between poor lung function and the increased risk of CVD. Previous studies showed that lung function was associated with mortality among smokers and non-smokers (4, 9). Inflammation, which leads to the degradation of lung function, might be the likely mechanism. Another potential mechanism that vascular injury and atherosclerosis owing to airflow limitation and mediating effects of chronic diseases on the association between lung function and mortality (10).

The incidence rate of type 2 diabetes mellitus has increased and became a global public health issue. Adults with diabetes are expected to surpass 700 million by 2025 (11). Diabetes is one of the most critical risk factors for CVD (12) and elevates morbidity and mortality of CVD mainly attributsed to vascular inflammation and endothelial dysfunction (13). Common complications of diabetes include microvascular and macrovascular conditions, such as retinopathy, nephropathy, neuropathy, cardiovascular, and peripheral vascular diseases (14). There is an increasing evidence that the lung is one of the target organs of diabetic damage (15). A meta-analysis reported that diabetes was associated with a decreased predicted percentage of forced expiratory volume in 1 s (FEV1%) and percentage of forced vital capacity (FVC%) (16). Reduced lung function is also associated with the risk of diabetes (8) and is often considered one of the complications of diabetes. Throughout the mechanisms of the association between lung function and CVD, systemic inflammation seems a common one, which contributes to the association of lung dysfunction with both CVD and diabetes. Besides, highly related characteristics between diabetes and CVD may also contaminate or exert effect modification on the association of lung dysfunction with CVD (8, 17). However, as an essential part of secondary prevention from cardiovascular mortality, the association between lung function and cardiovascular mortality among patients with diabetes has been neglected. The current study aimed to investigate whether reduced lung function is associated with the risk of all-cause and cardiovascular mortality among patients with diabetes by using participants in the Health Examination Survey (NHANES 1988–1994), which is a nationwide prospective cohort study.

## Methods

### Study population

**Figure 1:**
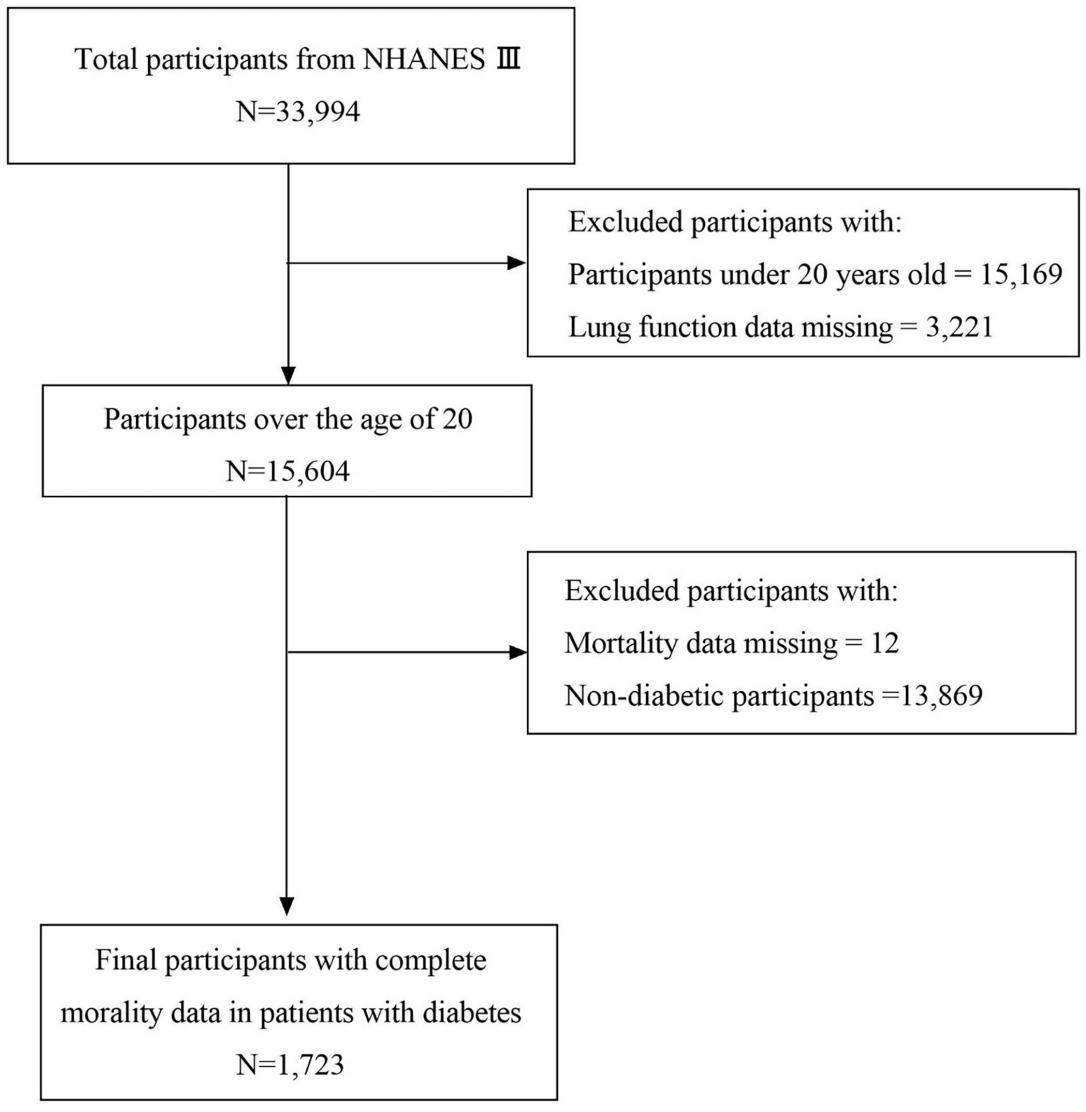
Study participant flowchart.

The participants were from the National Health and Nutrition Examination Survey (NHANES 1988–1994). The NHANES used a multi-stage, stratified, closeted, probability sampling design to identify a nationally representative sample of non-institutionalized civilians in the United States. The participants completed a household interview, laboratory measurements, and physical examinations. A detailed cohort profile was published previously (18). Data on the baseline lifestyle and participants' characteristics, including demographic data, medical history of related diseases, alcohol and smoking status, and other items, were compiled *via* a self-administered questionnaire. From 1988 to 1994, a total of 33,994 participants were enrolled in this study. In the current study, 18,390 participants were excluded owing to insufficient or missing spirometry data or being underage (age < 20 years), 13,869 were excluded without a history of diabetes, and 12 were excluded without mortality data A total of 1,723 individuals were included (760 men and 963 women) (Figure 1). The criteria of diabetic medical history are as follows: fasting plasma glucose >7.8 mmol/L; glycohemoglobin ≥ 6.5%; taking insulin or diabetic pills; and being told to have diabetes by a doctor. Hypertension was defined as being told by a doctor to have a high blood pressure. The definition of dyslipidemia was high-density lipoprotein cholesterol (HDL-C) < 40 mg/dl, as well as total cholesterol, low-density lipoprotein cholesterol (LDL-C), and TG levels of ≥200, ≥130, and ≥130 mg/dl, respectively.

NHANES is a publicly released dataset, so informed consent is not required.

### Lung function measurement

Lung function measurement was performed by following the standards (19) of the American Thoracic Society. Lung function parameters in the present study included FEV1, FVC, and FEV1/FVC. The FEV1 and FVC measurements were acquired from the spirometry data as part of the NHANES. Detailed information on the spirometry equipment, examination protocol, calibration procedures, and quality control for the NHANES was available and reported previously (20). FEV1 and FVC measurements were performed by trained technicians using a dry-rolling seal spirometry and involved the performance of at least five FVC maneuvers. Until it is accepted, it has to ensure a maneuver that is free of hesitation, leak, cough, mouthpiece obstruction, additional effort, and early termination. Only valid and reproducible spirometry measurements were chosen according to the reference values (21). The designated FEV1 and FVC values for each subject were obtained from the largest values of FEV1 and FVC, respectively, from the spirometry performed by each participant. We also calculated FEV1 and FVC as the percentage of predicted values for each participant according to Hankinson's predicted value equation (21).

### Mortality ascertainment

From its baseline (1988–1994) to the end of follow-up on 31 December 2015, participants' vital status and cause-of-death information were confirmed by the National Center for Health Statistics. Vital status was determined by the probabilistic matching of participants to the National Death Index based on identifying information, including social security number, name, sex, and date of birth (22). The identical matching methodology applied to the NHANES I Epidemiological Follow-up Study found that 96.1% of deceased participants and 99.4% of living participants were correctly classified (23). Details of the linkage methods have been reported previously (22). The International Classification of Disease, 10th revision (ICD10) codes, were applied to determine the underlying causes of death (24). In the present study, our primary outcome was the total CVD mortality (ICD I00-I09, I11, I13, I20-I51, I60-I69). Other cause-specific outcomes included mortalities from CHD (ICD I00-I09, I11, I13, I20-I51), cancer (C00-C97) and all-cause death. This death certificate ascertainment was applied to all deaths within our cohort, except for deaths with insufficient information on these matching criteria, which were considered as a lost follow-up.

### Statistical analysis

FEV1 and FVC were categorized by quartile. The significance of differences in means or proportions of participants' characteristics and risk factors of CVD and diabetes or covariates related to lung function was tested by covariance or χ^2^ test. Person-years of follow-up were calculated from the baseline (1988-1994) to their first endpoint in this follow-up as follows: death, moving out, or the end of follow-up, whichever came first. Adult participants in NHANES III were followed for mortality up to 31 December 2015. The Cox proportional hazard model was used to calculate crude and multivariable-adjusted hazard ratios (HRs) and 95% confidence intervals (95% CIs) for estimating the risk of mortality from CVD or all-cause mortality during the follow-up period across quartiles of FEV1 and FVC, respectively. Multiplicative interactions of FEV1 and FVC with sex were tested in deciding whether to present the data sex-specifically or to combine the results of men and women. We hypothesized known risk factors of CVD and covariates related to lung function as confounders, including sex, age, race, education level, BMI, drinking status, smoking status, high-density lipoprotein-cholesterol (HDL-c) level, serum C-reactive protein, serum albumin, the ratio of FEV1 and FVC, percentage of predicted values of FEV1 and FVC, history of hypertension and dyslipidemia, history of whistling and/or wheezing, persist phlegm status, asthma status, history of chronic bronchitis, and cold or flu. To avoid multicollinearity caused by multiple covariates, we conducted a multivariable Cox regression, including all covariates, and calculated the variance inflation factors (VIFs) as a diagnostic tool of multicollinearity. We assigned the median values to each quartile of FEV1 and FVC and examined their significance to calculate the trends across quartiles of FEV1 and FVC. Besides, we investigated the trends of FEV1 and FVC with increasing age and height by calculating the mean value and 95% CIs of FEV1 and FVC in different age or height groups.

Additionally, we conducted a stratified analysis according to BMI, age, history of hypertension, and dyslipidemia to examine the potential effect modification. In sensitivity analyses, we excluded those who died within 2 years to avoid potential as-yet-undiagnosed diseases at baseline, and who had a medical history of respiratory diseases (asthma, chronic bronchitis, and emphysema). All probability values for the statistical test were two-tailed, and *P* < 0.05 was considered statistically significant. Statistical analyses of the present study were conducted on the SAS statistical package (Version 9.4; SAS Inc., Cary, NC). NHANES recommends using sample weights to calculate estimates that represent the U.S. civilian non-institutionalized population or any subpopulation of interest. “PROC SURVEYREG” were used in computing descriptive and regression analyses as these protocols account for both the weighted data and the complexity of sample design.

## Results

Since no interaction with sex was observed in the association of FEV1 and FVC with CVD and specific endpoints, the presented results of men and women were combined in the main analyses. During the follow-up of 25,184 person-year of 1,723 included participants, 1,221 deaths were documented; 406 deaths due to CVD (327 of which were due to coronary heart disease) and 197 due to cancer.

In the multilinearity diagnosis, the highest VIF (1.56) occurred in the medical history of asthma, and no strong multilinearity was observed in the covariates (data not shown). In Table 1, participants in the lowest quartiles of FEV1 and FVC were older age, less likely to be current drinkers or smokers, had lower education levels, lower percentage of predicted values of FEV1 and FVC, lower serum albumin levels, higher HDL-c, and serum C-reactive protein level. They were also less likely to have a medical history of whistling or wheezing, and more likely to have a history of asthma, chronic bronchitis, emphysema, dyslipidemia, and hypertension.

**Table 1:** Baseline demographic characteristics of the study population, according to quartiles of FEV1, FVC.

**Characteristic**	**FEV1**					**FVC**				
	**Q1** **≥2883**	**Q2** **2307.5–2882.9**	**Q3** **1818–2307.4**	**Q4** **<1818**	**P-value**	**Q1** **≥3750.5**	**Q2** **2995.5–3750.4**	**Q3** **2379–2995.4**	**Q4** **<2379**	**P–value**
Total N	431	431	434	427		431	431	432	429	
**Age, y**					<0.001					<0.001
20–60	306(77.3)	221(59.5)	134(36.3)	65(16.4)		267(70.7)	212(57.9)	162(39.9)	85(23.5)	
≥60	125(22.7)	210(40.6)	300(63.7)	362(83.6)		164(29.3)	219(42.1)	270(60.1)	344(76.5)	
**Gender**					<0.001					<0.001
Men	343(78.8)	220(42.5)	110(26.0)	87(20.5)		377(84.9)	233(44.4)	97(18.9)	53(12.8)	
Women	88(21.3)	211(57.5)	324(74.0)	340(79.5)		54(15.1)	198(55.6)	335(81.1)	376(87.2)	
**Race/ethnicity**					<0.001					<0.001
Non–Hispanic white	146(73.5)	143(71.8)	150(63.4)	157(62.7)		176(79.0)	139(69.0)	136(65.0)	145(54.9)	
Non–Hispanic black	109(12.1)	132(15.7)	143(20.7)	156(23.4)		100(10.3)	133(16.3)	139(19.1)	168(27.9)	
Mexican American	166(8.6)	145(7.2)	124(6.3)	100(4.8)		147(6.8)	148(7.7)	139(7.8)	101(5.5)	
Other	10(5.8)	11(5.2)	17(9.6)	14(9.1)		8(3.9)	11(7.0)	18(8.1)	15(11.7)	
**Education, year**	10.25 ± 4.07	9.66 ± 4.31	8.91 ± 4.27	8.03 ± 4.25	<0.001	10.15 ± 4.2	9.71 ± 4.17	8.74 ± 4.28	8.25 ± 4.3	<0.001
**BMI, kg/m** ^**2**^					0.034					0.152
<25.0 (Normal)	78(16.5)	71(15.9)	88(23.6)	95(25.7)		89(16.3)	81(20.0)	76(19.9)	86(25.0)	
25.0–29.9 (Overweight)	153(34.5)	186(37.8)	144(30.0)	149(31.0)		166(36.2)	173(34.9)	156(34.8)	137(26.3)	
≥30.0 (Obese)	200(49.0)	174(46.3)	200(46.4)	181(43.3)		176(47.5)	176(45.1)	198(45.3)	205(48.7)	
**Alcohol**					<0.001					<0.001
Never drinker	354(82.1)	377(87.5)	385(91.5)	364(94.0)		347(81.6)	378(85.8)	385(93.5)	370(95.5)	
Moderate drinking	33(7.1)	15(7.0)	11(3.0)	6(2.4)		37(8.9)	16(6.2)	8(1.7)	4(1.7)	
Heavy drinking	34(10.8)	24(5.5)	13(5.5)	12(3.7)		34(9.5)	27(8.0)	12(4.8)	10(2.8)	
**Smoking**					<0.001					<0.001
Never smoker	148(34.1)	190(41.7)	221(45.9)	238(45.6)		119(27.6)	189(44.1)	220(43.5)	269(56.8)	
Former smoker	180(43.4)	161(36.3)	149(37.0)	121(33.8)		203(47.8)	157(36.9)	145(36.2)	106(25.8)	
Current smoker	103(22.5)	80(22.0)	64(17.1)	68(20.5)		109(24.6)	85(19.0)	67(20.3)	54(17.4)	
Fev1%pred	1.01 ± 0.13	0.94 ± 0.13	0.92 ± 0.17	0.74 ± 0.23	<0.001	0.99 ± 0.14	0.93 ± 0.16	0.91 ± 0.18	0.78 ± 0.23	<0.001
Fvc%pred	1.01 ± 0.12	0.94 ± 0.13	0.93 ± 0.16	0.77 ± 0.20	<0.001	1.02 ± 0.12	0.95 ± 0.13	0.91 ± 0.16	0.76 ± 0.20	<0.001
Fev1/Fvc	0.79 ± 0.06	0.78 ± 0.08	0.77 ± 0.09	0.73 ± 0.15	<0.001	0.76 ± 0.09	0.77 ± 0.09	0.77 ± 0.09	0.78 ± 0.13	<0.001
**HDL–cholesterol (mg/dL)**	42.89 ± 12.81	46.54 ± 14.08	48.38 ± 15.53	49.57 ± 16.09	<0.001	43.07 ± 12.86	45.65 ± 13.19	48.44 ± 15.49	50.21 ± 16.72	<0.001
Serum C–reactive protein (mg/dL)	0.5 ± 0.58	0.74 ± 1.19	0.79 ± 1.07	0.91 ± 1.13	<0.001	0.46 ± 0.61	0.73 ± 1.03	0.82 ± 1.09	0.92 ± 1.23	<0.001
Serum albumin (g/dL)	4.12 ± 0.39	4 ± 0.36	3.98 ± 0.36	3.91 ± 0.42	<0.001	4.13 ± 0.37	3.99 ± 0.39	3.98 ± 0.36	3.90 ± 0.40	<0.001
**History of whistling and/or wheezing**					<0.001					0.004
No	55(17.0)	67(18.6)	81(21.9)	102(27.4)		53(14.5)	76(23.7)	86(25.5)	90(21.3)	
Yes	376(83.0)	364(81.4)	353(78.1)	325(72.6)		378(85.5)	355(76.3)	346(74.5)	339(78.7)	
**Persist phlegm**					0.309					0.210
Yes	34(10)	44(8.5)	42(11.5)	50(12.4)		40(9.8)	52(12.9)	34(8.1)	44(10.8)	
No	397(90.0)	387(91.5)	391(88.5)	377(87.6)		391(90.2)	379(87.2)	398(91.9)	384(89.2)	
**Persist cough**					0.212					0.372
Yes	29(6.8)	36(11.9)	38(10.5)	46(14.4)		36(7.3)	39(14.5)	30(7.8)	44(13.5)	
No	402(93.2)	395(88.1)	396(89.5)	381(85.6)		395(92.7)	392(85.5)	402(92.2)	385(86.5)	
History of asthma, %	16(5.6)	252(7.7)	25(9.2)	60(13.7)	<0.001	20(6.6)	28(9.1)	31(9.8)	47(9.7)	<0.001
History of chronic bronchitis, %	20(7.4)	35(8.6)	38(13.3)	62(18.5)	<0.001	20(7.7)	43(11.4)	38(14.3)	54(13.7)	<0.001
fHistory of emphysema, %	4(2.1)	11(2.6)	13(4.7)	29(9.4)	<0.001	8(2.6)	18(5.6)	13(5.0)	18(4.6)	0.169
**History of clod or flu/year**					<0.001					0.066
No	157(30.0)	166(36.7)	194(50.4)	203(49.4)		161(33.7)	169(33.8)	187(48.2)	203(50.0)	
1time	156(41.8)	164(39.5)	147(30.6)	132(32.6)		163(41.9)	155(38.2)	144(32.2)	137(32.0)	
≥2times	117(28.2)	100(23.8)	91(19.0)	86(18.0)		106(24.4)	106(28.0)	98(19.5)	84(18.0)	
Dyslipidemia, %	361(80.4)	361(86.0)	394(93.9)	379(90.1)	0.002	361(81.3)	362(85.6)	388(92.3)	384(91.2)	0.006
History of hypertension,%	192(46.0)	206(46.1)	242(57.0)	255(59.9)	<0.001	193(46.0)	213(48.2)	226(56.5)	263(58.1)	<0.001

CVD, cardiovascular disease; CHD, coronary heart disease; BMI, body mass index; FEV1, forced expiratory volume in 1 s; FVC, forced vital capacity; HDL, high–density lipoprotein cholesterol.

In Table 2, both lower FEV1 and FVC were associated with a higher risk of CHD and CVD death. Compared with the highest group, HRs (95%CIs) of the lowest FEV1 were 2.71 (1.15–6.38; P for trend = 0.009) for CHD and 2.89 (1.19–3.87; P for trend = 0.001) for CVD mortality. HRs (95%CIs) of the lowest FVC were 5.30 (2.68-10.47; *P* for trend <0.001) for CHD and 6.32 (3.19–12.53; *P* for trend <0.001) for CVD. Although the lowest FVC was associated with a higher risk of cancer mortality (HR: 3.85 (1.31-11.32); *P* for trend = 0.040), the association of FEV1 was not related after adjustment for covariates (HR:2.23 (0.54–9.17); *P* for trend = 0.247). In addition, the lowest FEV1 and FVC were observed to be associated with all-cause deaths; HRs (95%CIs) of FEV1 and FVC were 2.96 (1.92–4.56; *P* for trend <0.001) and 4.15 (2.58–6.65; *P* for trend <0.001), respectively.

**Table 2:** Associations of FEV1 and FVC with coronary heart disease, cardiovascular, cancer and all–cause mortality in U.S. adults aged at least 20 years.

	**FEV1**					**FVC**				
	**Q1** **≥2883**	**Q2** **2307.5–2882.9**	**Q3** **1818–2307.4**	**Q4** **<1818**	***P for trend***	**Q1** **≥3750.5**	**Q2 2995.5–3750.4**	**Q3** **2379–2995.4**	**Q4 <2379**	***P for trend***
**CHD mortality**										
Deaths, no. (%)	54(12.6)	69(14.0)	97(26.9)	107(22.8)	<0.001	58(13.2)	77(16.8)	85(22.2)	107(23.4)	<0.001
Deaths/person–years	689/7810	833/7102	840/5966	839/4306		705/7416	790/6834	866/6412	840/4522	
Unadjusted	1.00 [Reference]	1.15(0.59,2.25)	2.95(1.83,4.75)	3.46(2.17,5.52)	<0.001	1.00 [Reference]	1.36(0.75,2.46)	2.05(1.21,3.47)	3.06(1.90,4.92)	<0.001
Model 1	1.00 [Reference]	1.09(0.60,2.01)	2.47(1.44,4.22)	2.41(1.27,4.56)	0.003	1.00 [Reference]	1.54(0.89,2.67)	2.23(1.21,4.13)	2.84(1.36,5.94)	0.006
Model 2	1.00 [Reference]	1.06(0.58,1.95)	2.31(1.39,3.84)	2.23(1.21,4.11)	0.005	1.00 [Reference]	1.66(0.97,2.86)	2.22(1.25,3.93)	2.82(1.37,5.80)	0.005
Model 3	1.00 [Reference]	1.09(0.54,2.19)	2.40(1.29,4.46)	2.59(1.06,6.34)	0.015	1.00 [Reference]	1.92(1.11,3.32)	2.89(1.58,5.25)	4.54(2.02,10.20)	<0.001
Model 4	1.00 [Reference]	1.11(0.54,2.25)	2.46(1.34,4.51)	2.71(1.15,6.38)	0.009	1.00 [Reference]	2.05(1.24,3.40)	2.96(1.63,5.37)	5.30(2.68,10.47)	<0.001
**CVD mortality**										
Deaths, no. (%)	68(15.0)	85(17.1)	122(32.9)	131(26.4)	<0.001	74(15.4)	91(20.2)	108(27.3)	133(28.0)	<0.001
Deaths/person–years	862/7810	977/7102	1165/5966	1028/4306		884/7416	932/6834	1148/6412	1068/4522	
Unadjusted	1.00 [Reference]	1.19(0.65,2.17)	3.05(1.95,4.77)	3.38(2.21,5.15)	<0.001	1.00 [Reference]	1.40(0.84,2.34)	2.15(1.33,3.48)	3.13(2.07,4.74)	<0.001
Model 1	1.00 [Reference]	1.08(0.61,1.92)	2.36(1.45,3.85)	2.17(1.22,3.88)	0.003	1.00 [Reference]	1.54(0.93,2.56)	2.19(1.24,3.89)	2.72(1.41,5.26)	0.003
Model 2	1.00 [Reference]	1.08(0.61,1.89)	2.30(1.46,3.63)	2.15(1.19,3.87)	0.003	1.00 [Reference]	1.74(1.02,2.98)	2.31(1.36,3.94)	2.96(1.47,5.95)	0.002
Model 3	1.00 [Reference]	1.15(0.61,2.16)	2.57(1.52,4.32)	2.75(1.25,6.02)	0.003	1.00 [Reference]	2.14(1.24,3.70)	3.29(1.87,5.78)	5.45(2.48,11.97)	<0.001
Model 4	1.00 [Reference]	1.19(0.65,2.19)	2.70(1.61,4.53)	2.89(1.36,6.18)	0.001	1.00 [Reference]	2.28(1.39,3.76)	3.40(1.96,5.89)	6.32(3.19,12.53)	<0.001
**Cancer mortality**										
Deaths, no. (%)	35(7.0)	51(10.7)	59(14.6)	52(12.2)	0.073	42(9.9)	57(10.7)	51(10.5)	47(12.0)	0.434
Deaths/person–years	385/7810	579/7102	569/5966	429/4306		426/7416	622/6834	528/6412	385/4522	
Unadjusted	1.00 [Reference]	1.58(0.79,3.17)	2.86(1.60,5.14)	3.34(1.79,6.23)	<0.001	1.00 [Reference]	1.14(0.54,2.41)	1.28(0.63,2.62)	2.10(1.15,3.84)	0.052
Model 1	1.00 [Reference]	1.45(0.66,3.18)	2.29(0.89,5.87)	2.19(0.83,5.79)	0.079	1.00 [Reference]	1.14(0.51,2.53)	1.11(0.43,2.85)	1.51(0.63,3.64)	0.402
Model 2	1.00 [Reference]	1.43(0.66,3.10)	2.05(0.76,5.50)	2.07(0.73,5.88)	0.139	1.00 [Reference]	1.25(0.58,2.72)	1.03(0.41,2.64)	1.61(0.64,4.05)	0.383
Model 3	1.00 [Reference]	1.37(0.57,3.27)	1.97(0.56,6.96)	2.01(0.43,9.48)	0.314	1.00 [Reference]	1.63(0.72,3.73)	1.51(0.48,4.73)	3.13(1.02,9.55)	0.086
Model 4	1.00 [Reference]	1.37(0.61,3.06)	1.91(0.59,6.23)	2.23(0.54,9.17)	0.247	1.00 [Reference]	1.74(0.74,4.12)	1.53(0.45,5.27)	3.85(1.31,11.32)	0.040
**All–cause mortality**										
Deaths, no. (%)	230(49.5)	277(56.4)	332(76.9)	382(89.5)	<0.001	258(54.4)	281(58.3)	314(72.9)	368(84.3)	<0.001
Deaths/person–years	3015/7810	3445/7102	3604/5966	3316/4306		3277/7416	3256/6834	3659/6412	3188/4522	
Unadjusted	1.00 [Reference]	1.18(0.87,1.61)	2.24(1.69,2.97)	3.78(2.88,4.95)	<0.001	1.00 [Reference]	1.14(0.87,1.51)	1.67(1.23,2.26)	2.87(2.10,3.92)	<0.001
Model 1	1.00 [Reference]	1.14(0.88,1.48)	1.91(1.34,2.71)	2.70(1.77,4.12)	<0.001	1.00 [Reference]	1.28(0.99,1.65)	1.74(1.21,2.50)	2.57(1.58,4.20)	<0.001
Model 2	1.00 [Reference]	1.12(0.87,1.44)	1.76(1.30,2.38)	2.51(1.69,3.75)	<0.001	1.00 [Reference]	1.37(1.05,1.77)	1.67(1.23,2.28)	2.62(1.59,4.32)	<0.001
Model 3	1.00 [Reference]	1.17(0.85,1.60)	1.89(1.32,2.72)	3.01(1.87,4.86)	<0.001	1.00 [Reference]	1.56(1.17,2.08)	2.11(1.48,2.99)	3.92(2.24,6.85)	<0.001
Model 4	1.00 [Reference]	1.15(0.85,1.55)	1.81(1.29,2.55)	2.96(1.92,4.56)	<0.001	1.00 [Reference]	1.59(1.18,2.12)	2.08(1.49,2.92)	4.15(2.58,6.65)	<0.001

Values are n or weighted hazard ratio (95% confidence interval).

Model 1: adjusted for sex, age.

Model 2: model 1 + education, BMI, alcohol, and smoking.

Model 3: model 2 + HDL–cholesterol, serum C–reactive protein, serum albumin, fev1/fvc and fev1%pred or fvc% pred.

Model 4: model 3 + history of hypertension, history of dyslipidemia, history of whistling and/or wheezing, persist phlegm, persist cough, asthma, history of chronic bronchitis, history of emphysema, and history of cold or flu.

CHD, coronary heart disease.

CVD, cardiovascular disease.

BMI, body mass index.

FEV1, forced expiratory volume in 1 s.

FVC, forced vital capacity.

As shown in Tables 3.1–3.4, we conducted stratified analyses based on BMI, age, history of hypertension, and dyslipidemia. Among participants with BMI of ≥25, lower FEV1 was associated with a higher risk of CHD, CVD, and all-cause mortality; HRs (95% CIs) were 2.91(1.19–7.10), 3.19 (1.40–7.24), and 3.86 (2.12–7.03), respectively, but only associated with CHD and CVD among the participants whose BMI was <25. For FVC, the associations with CHD, CVD, and all-cause death were observed in both subgroups of BMI. The association of FEV1 was observed with all-cause mortality in both subgroups stratified by age. On the other hand, the association of FEV1 with CVD was only observed in the subgroup aged ≥60 (HR:3.61; 95%CI: 1.23–10.58). For FVC, the associations were significant in both subgroups with CHD, CVD, and all-cause mortality.

**Table 3.1:** Associations of FEV1 and FVC with coronary heart disease mortality in U.S. adults aged at least 20 years among different groups.

	**FEV1**					**FVC**				
	**Q1** **≥2883**	**Q2** **2307.5–2882.9**	**Q3** **1818–2307.4**	**Q4** **<1818**	***P for trend***	**Q1** **≥3750.5**	**Q2** **2995.5–3750.4**	**Q3** **2379–2995.4**	**Q4** **<2379**	***P for trend***
**CHD mortality**										
**Age**										
**≥60**	*N =* 997									
Deaths/N	26/125	49/210	85/300	100/362	0.276	34/164	57/219	71/270	98/344	0.324
Multivariable HR (95% CI)	1.00 [Reference]	1.17(0.48,2.83)	2.28(0.91,5.69)	2.68(0.80,8.93)	0.063	1.00 [Reference]	2.49(1.46,4.25)	2.45(1.15,5.21)	4.14(1.90,9.03)	0.001
**<60**	*N =* 726									
Deaths/N	28/306	20/221	12/134	7/65	0.977	24/267	20/212	14/162	9/85	
Multivariable HR (95% CI)	1.00 [Reference]	1.07(0.34,3.41)	3.06(0.91,10.31)	2.90(0.42,19.99)	0.155	1.00 [Reference]	1.72(0.43,6.88)	6.31(1.36,29.32)	10.33(1.22,87.67)	0.028
*P for interaction*	0.416					0.503				
**BMI**										
**≥25**	*N =* 1387									
Deaths/N	44/353	57/360	66/344	75/330	0.003	45/342	56/349	65/354	76/342	0.015
Multivariable HR (95% CI)	1.00 [Reference]	1.49(0.78,2.86)	2.79(1.44,5.41)	2.91(1.19,7.10)	0.007	1.00 [Reference]	2.37(1.23,4.57)	3.60(1.78,7.32)	5.34(2.26,12.61)	<0.001
**<25**	*N =* 332									
Deaths/N	10/78	12/71	31/88	31/95	0.001	13/89	21/81	19/76	31/86	0.014
Multivariable HR (95% CI)	1.00 [Reference]	0.22(0.05,1.04)	2.38(0.57,9.83)	3.65(0.78,17.09)	0.045	1.00 [Reference]	1.19(0.31,4.65)	4.05(0.99,16.48)	14.01(2.38,82.62)	0.013
*P for interaction*	0.138					0.116				
**History of hypertension**										
**Yes**	*N =* 895									
Deaths/N	33/192	38/206	55/242	63/255	0.171	35/193	47/213	44/226	63/263	0.426
Multivariable HR (95% CI)	1.00 [Reference]	1.66(0.74,3.73)	3.38(1.46,7.85)	3.87(1.21,12.40)	0.010	1.00 [Reference]	1.70(0.85,3.43)	2.26(0.85,5.98)	3.89(1.47,10.32)	0.011
**No**	*N =* 823									
Deaths/N	21/238	31/224	42/191	44/170	<0.001	23/237	30/217	41/205	44/164	<0.001
Multivariable HR (95% CI)	1.00 [Reference]	0.75(0.28,2.01)	1.65(0.71,3.86)	2.25(0.61,8.29)	0.158	1.00 [Reference]	2.34(1.15,4.76)	3.98(2.24,7.07)	8.91(3.76,21.13)	<0.001
*P for interaction*	0.248					0.143				
**Dyslipidemia**										
**Yes**	*N =* 1495									
Deaths/N	48/361	54/361	91/394	92/379	<0.001	48/361	63/362	78/388	96/384	<0.001
Multivariable HR (95% CI)	1.00 [Reference]	0.90(0.42,1.93)	2.17(1.18,3.98)	2.29(0.95,5.51)	0.023	1.00 [Reference]	1.86(1.04,3.35)	2.89(1.57,5.32)	5.37(2.63,10.95)	<0.001
**No**	*N =* 228									
Deaths/N	6/70	15/70	6/40	15/48	0.015	10/70	14/69	7/44	11/45	0.526
Multivariable HR (95% CI)	1.00 [Reference]	3.00(0.49,18.31)	2.90(0.34,24.54)	12.79(0.84,194.24)	0.070	1.00 [Reference]	2.10(0.33,13.45)	1.45(0.20,10.45)	5.55(0.57,53.78)	0.145
*P for interaction*	0.139					0.509				

Multivariable Model: adjusted for sex, age, education, BMI, alcohol, and smoking, HDL–cholesterol, serum C–reactive protein, serum albumin, fev1/fvc and fev1% pred or fvc% pred, history of hypertension and history of dyslipidemia, history of whistling and/or wheezing, persist phlegm, persist cough, asthma, history of chronic bronchitis, history of emphysema, and history of cold or flu.

CHD, coronary heart disease.

CVD, cardiovascular disease.

BMI, body mass index.

FEV1, forced expiratory volume in 1 s.

FVC, forced vital capacity.

**Table 3.2:** Associations of FEV1 and FVC with cardiovascular mortality in U.S. adults aged at least 20 years among different groups.

	**FEV1**					**FVC**				
	**Q1** **≥2883**	**Q2** **2307.5–2882.9**	**Q3** **1818–2307.4**	**Q4** **<1818**	***P for trend***	**Q1** **≥3750.5**	**Q2** **2995.5–3750.4**	**Q3** **2379–2995.4**	**Q4** **<2379**	***P for trend***
**CVD mortality**										
**Age**										
**≥60**	*N =* 997									
Deaths/N	34/125	61/210	105/300	123/362	0.266	45/164	68/219	87/270	123/344	0.001
Multivariable HR (95% CI)	1.00 [Reference]	1.50(0.66,3.41)	3.04(1.35,6.88)	3.61(1.23,10.58)	0.007	1.00 [Reference]	3.04(1.70,5.44)	3.22(1.60,6.48)	5.88(2.71,12.76)	<0.001
**<60**	*N =* 726									
Deaths/N	34/306	24/221	17/134	8/65	0.948	29/267	23/212	21/162	10/85	0.910
Multivariable HR (95% CI)	1.00 [Reference]	0.96(0.34,2.70)	2.67(0.88,8.12)	2.45(0.39,15.49)	0.206	1.00 [Reference]	1.93(0.61,6.06)	6.09(1.50,24.65)	10.31(1.59,66.87)	0.014
*P for interaction*	0.694					0.810				
**BMI**										
**≥25**	*N =* 1387									
Deaths/N	56/353	69/360	86/344	95/330	<0.001	57/342	68/349	82/354	99/342	<0.001
Multivariable HR (95% CI)	1.00 [Reference]	1.55(0.85,2.85)	3.01(1.69,5.34)	3.19(1.40,7.24)	0.001	1.00 [Reference]	2.76(1.46,5.22)	4.28(2.28,8.03)	1.01(3.19,15.39)	<0.001
**<25**	*N =* 332									
Deaths/N	12/78	16/71	36/88	35/95	0.001	17/89	23/81	25/76	34/86	0.027
Multivariable HR (95% CI)	1.00 [Reference]	0.27(0.06,1.15)	2.77(0.74,10.44)	3.35(0.80,14.02)	0.027	1.00 [Reference]	1.10(0.29,4.21)	3.64(1.04,12.74)	10.23(1.93,54.24)	0.017
*P for interaction*	0.138					0.116				
**History of hypertension**										
**Yes**	*N =* 895									
Deaths/N	44/192	46/206	76/242	80/255	0.036	47/193	56/213	61/226	82/263	0.403
Multivariable HR (95% CI)	1.00 [Reference]	1.61(0.72,3.60)	3.88(1.75,8.57)	4.30(1.57,11.79)	0.002	1.00 [Reference]	1.97(0.89,4.36)	3.10(1.22,7.85)	5.91(2.21,15.79)	0.001
**No**	*N =* 823									
Deaths/N	23/238	39/224	46/191	51/170	<0.001	26/237	35/217	47/205	51/164	<0.001
Multivariable HR (95% CI)	1.00 [Reference]	0.85(0.35,2.05)	1.54(0.68,3.49)	2.15(0.62,7.41)	0.196	1.00 [Reference]	2.66(1.34,5.30)	4.05(2.37,6.92)	8.77(3.46,22.25)	<0.001
*P for interaction*	0.213					0.116				
**Dyslipidemia**										
**Yes**	*N =* 1495									
Deaths/N	61/361	66/361	113/394	115/379	<0.001	62/361	75/362	98/388	120/384	<0.001
Multivariable HR (95% CI)	1.00 [Reference]	1.02(0.52,1.99)	2.58(1.54,4.33)	2.82(1.29,6.16)	0.002	1.00 [Reference]	2.25(1.25,4.04)	3.49(1.97,6.19)	7.04(3.37,14.69)	<0.001
**No**	*N =* 228									
Deaths/N	7/70	19/70	9/40	16/48	0.015	12/70	16/69	10/44	13/45	0.526
Multivariable HR (95% CI)	1.00 [Reference]	2.82(0.74,10.71)	1.94(0.27,14.06)	5.93(0.54,64.57)	0.238	1.00 [Reference]	1.91(0.49,7.47)	2.38(0.45,12.57)	4.26(0.89,20.44)	0.084
*P for interaction*	0.218						0.667			

Multivariable Model: adjusted for sex, age, education, BMI, alcohol, and smoking, HDL–cholesterol, serum C–reactive protein, serum albumin, fev1/fvc and fev1%pred or fvc%pred, history of hypertension and history of dyslipidemia, history of whistling and/or wheezing, persist phlegm, persist cough, asthma, history of chronic bronchitis, history of emphysema, and history of cold or flu.

CHD, coronary heart disease.

CVD, cardiovascular disease.

BMI, body mass index.

FEV1, forced expiratory volume in 1 s.

FVC, forced vital capacity.

**Table 3.3:** Associations of FEV1 and FVC with cancer mortality in U.S. adults aged at least 20 years among different groups.

	**FEV1**					**FVC**				
	**Q1** **≥2883**	**Q2** **2307.5–2882.9**	**Q3** **1818–2307.4**	**Q4** **<1818**	***P for trend***	**Q1** **≥3750.5**	**Q2** **2995.5–3750.4**	**Q3** **2379–2995.4**	**Q4** **<2379**	***P for trend***
**Cancer mortality**										
**Age**										
**≥60**	*N =* 997									
Deaths/N	20/125	33/210	48/300	41/362	0.265	15/306	18/221	11/134	11/65	0.036
Multivariable HR (95% CI)	1.00 [Reference]	1.08(0.42,2.77)	1.25(0.33,4.67)	1.37(0.26,7.16)	0.697	1.00 [Reference]	2.22(0.73,6.76)	3.34(0.86,12.99)	5.50(0.56,54.33)	0.085
**<60**	*N =* 726									
Deaths/N	15/306	18/221	11/134	11/65	0.010a	13/267	17/212	14/162	11/85	0.085
Multivariable HR (95% CI)	1.00 [Reference]	1.32(0.45,3.89)	0.74(0.17,3.23)	2.01(0.53,7.60)	0.462	1.00 [Reference]	3.01(0.71,12.74)	7.16(1.13,45.29)	8.86(0.74,106.8)	0.047
*P for interaction*	0.033					0.070				
**BMI**										
**≥25**	*N =* 1387									
Deaths/N	29/353	41/360	41/344	40/330	0.310	33/342	46/349	37/354	35/342	0.499
Multivariable HR (95% CI)	1.00 [Reference]	1.54(0.67,3.54)	1.82(0.62,5.39)	2.69(0.66,10.99)	0.178	1.00 [Reference]	1.48(0.55,4.01)	1.34(0.36,4.97)	2.71(0.69,10.62)	0.216
**<25**	*N =* 332									
Deaths/N	6/78	10/71	18/88	12/95	0.121	9/89	11/81	14/76	12/86	0.498
Multivariable HR (95% CI)	1.00 [Reference]	1.18(0.39,3.55)	1.42(0.17,12.11)	0.87(0.07,11.54)	0.968	1.00 [Reference]	3.84(1.41,10.5)	1.65(0.27,10.29)	15.03(3.54,63.75)	0.061
*P for interaction*	0.741					0.196				
**History of hypertension**										
**Yes**	*N =* 895									
Deaths/N	15/192	26/206	37/242	38/255	0.088	16/193	34/213	33/226	33/263	0.111
Multivariable HR (95% CI)	1.00 [Reference]	1.19(0.35,4.09)	1.99(0.47,8.41)	2.09(0.31,13.94)	0.354	1.00 [Reference]	2.22(0.84,5.89)	1.65(0.44,6.15)	5.24(1.77,15.55)	0.005
**No**	*N =* 823									
Deaths/N	20/238	25/224	22/191	14/170	0.553	26/237	23/217	18/205	14/164	0.788
Multivariable HR (95% CI)	1.00 [Reference]	1.77(0.84,3.70)	2.27(0.60,8.55)	2.54(0.46,13.95)	0.205	1.00 [Reference]	1.86(0.62,5.63)	2.24(0.41,12.26)	3.03(0.37,24.69)	0.276
*P for interaction*	0.057					0.098				
**Dyslipidemia**										
**Yes**	*N =* 1495									
Deaths/N	29/361	43/361	54/394	51/379	0.064	33/361	48/362	49/388	47/384	0.320
Multivariable HR (95% CI)	1.00 [Reference]	1.55(0.68,3.51)	2.11(0.73,6.14)	2.84(0.76,10.58)	0.119	1.00 [Reference]	1.90(0.76,4.77)	1.99(0.60,6.54)	5.46(1.92,15.53)	0.008
**No**	*N =* 228									
Deaths/N	6/70	8/70	5/40	1/48	0.262	9/70	9/69	2/44	0/45	0.039
Multivariable HR (95% CI)	–	–	–	–		–	–	–	–	
*P for interaction*	0.245					0.357				

Multivariable Model: adjusted for sex, age, education, BMI, alcohol, and smoking, HDL–cholesterol, serum C–reactive protein, serum albumin, fev1/fvc and fev1%pred or fvc%pred, history of hypertension and history of dyslipidemia, history of whistling and/or wheezing, persist phlegm, persist cough, asthma, history of chronic bronchitis, history of emphysema, and history of cold or flu.

CHD, coronary heart disease.

CVD, cardiovascular disease.

BMI, body mass index.

FEV1, forced expiratory volume in 1 s.

FVC, forced vital capacity.

**Table 3.4:** Associations of FEV1 and FVC with all–cause mortality in U.S. adults aged at least 20 years among different groups.

	**FEV1**					**FVC**				
	**Q1** **≥2883**	**Q2** **2307.5–2882.9**	**Q3** **1818–2307.4**	**Q4** **<1818**	***P for trend***	**Q1** **≥3750.5**	**Q2** **2995.5–3750.4**	**Q3** **2379–2995.4**	**Q4** **<2379**	***P for trend***
**All–cause mortality**										
**Age**										
**≥60**	*N =* 997									
Deaths/N	106/125	187/210	269/300	340/362	0.016	144/164	198/219	241/270	319/344	0.278
Multivariable HR (95% CI)	1.00 [Reference]	1.21(0.82,1.80)	1.71(1.01,2.87)	3.01(1.70,5.32)	<0.001	1.00 [Reference]	1.79(1.30,2.46)	1.84(1.20,2.83)	3.8(2.24,6.46)	<0.001
**<60**	*N =* 726									
Deaths/N	124/306	90/221	63/134	42/65	0.003	114/267	83/212	73/162	49/85	0.033
Multivariable HR (95% CI)	1.00 [Reference]	1.20(0.76,1.87)	1.88(0.99,3.52)	2.61(1.12,6.12)	0.047	1.00 [Reference]	1.56(0.80,3.02)	2.98(1.46,6.10)	5.15(1.88,14.14)	0.002
*P for interaction*	0.416					0.241				
**BMI**										
**≥25**	*N =* 1387									
Deaths/N	182/353	226/360	250/344	290/330	<0.001	197/342	216/349	248/354	287/342	<0.001
Multivariable HR (95% CI)	1.00 [Reference]	1.55(1.13,2.12)	2.20(1.46,3.32)	3.86(2.12,7.03)	<0.001	1.00 [Reference]	1.40(1.01,1.93)	2.11(1.43,3.11)	3.44(1.98,5.99)	0.001
**<25**	*N =* 332									
Deaths/N	48/78	51/71	81/88	90/95	<0.001	61/89	64/81	65/76	80/86	<0.001
Multivariable HR (95% CI)	1.00 [Reference]	0.49(0.24,1.01)	1.01(0.47,2.16)	1.82(0.71,4.65)	0.111	1.00 [Reference]	1.97(0.94,4.12)	2.40(1.15,50)	7.69(2.90,20.44)	<0.001
*P for interaction*	0.064					0.043				
**History of hypertension**										
**Yes**	*N =* 895									
Deaths/N	115/192	145/206	192/242	231/255	<0.001	128/193	153/213	173/226	229/263	<0.001
Multivariable HR (95% CI)	1.00 [Reference]	1.28(0.80,2.06)	1.90(1.21,2.98)	3.24(1.79,5.86)	<0.001	1.00 [Reference]	1.34(0.89,2.01)	1.83(1.19,2.79)	3.69(2.16,6.29)	<0.001
**No**	*N =* 823									
Deaths/N	114/238	132/224	140/191	149/170	<0.001	129/237	128/217	141/205	137/164	<0.001
Multivariable HR (95% CI)	1.00 [Reference]	1.04(0.65,1.65)	1.66(1.06,2.59)	2.67(1.30,5.47)	0.011	1.00 [Reference]	1.81(1.26,2.61)	2.38(1.46,3.90)	4.69(2.29,9.61)	<0.001
*P for interaction*	0.410					0.372				
**Dyslipidemia**										
**Yes**	*N =* 1495									
Deaths/N	198/361	233/361	305/394	341/379	<0.001	220/361	239/362	287/388	331/384	<0.001
Multivariable HR (95% CI)	1.00 [Reference]	1.14(0.85,1.53)	1.88(1.40,2.52)	3.31(2.20,4.98)	<0.001	1.00 [Reference]	1.59(1.22,2.06)	2.21(1.63,2.99)	4.70(3.17,6.97)	<0.001
**No**	*N =* 228									
Deaths/N	32/70	44/70	27/40	41/48	<0.001	38/70	42/69	27/44	37/45	0.022
Multivariable HR (95% CI)	1.00 [Reference]	0.68(0.24,1.93)	1.01(0.27,3.74)	1.04(0.19,5.58)	0.905	1.00 [Reference]	0.79(0.30,2.05)	1.30(0.32,5.30)	1.79(0.39,8.27)	0.567
*P for interaction*	0.911					0.475				

Multivariable Model: adjusted for sex, age, education, BMI, alcohol, and smoking, HDL–cholesterol, serum C–reactive protein, serum albumin, fev1/fvc and fev1% pred or fvc% pred, history of hypertension and history of dyslipidemia, history of whistling and/or wheezing, persist phlegm, persist cough, asthma, history of chronic bronchitis, history of emphysema, and history of cold or flu.

CHD, coronary heart disease.

CVD, cardiovascular disease.

BMI, body mass index.

FEV1, forced expiratory volume in 1 s.

FVC, forced vital capacity.

**Figure 2:**
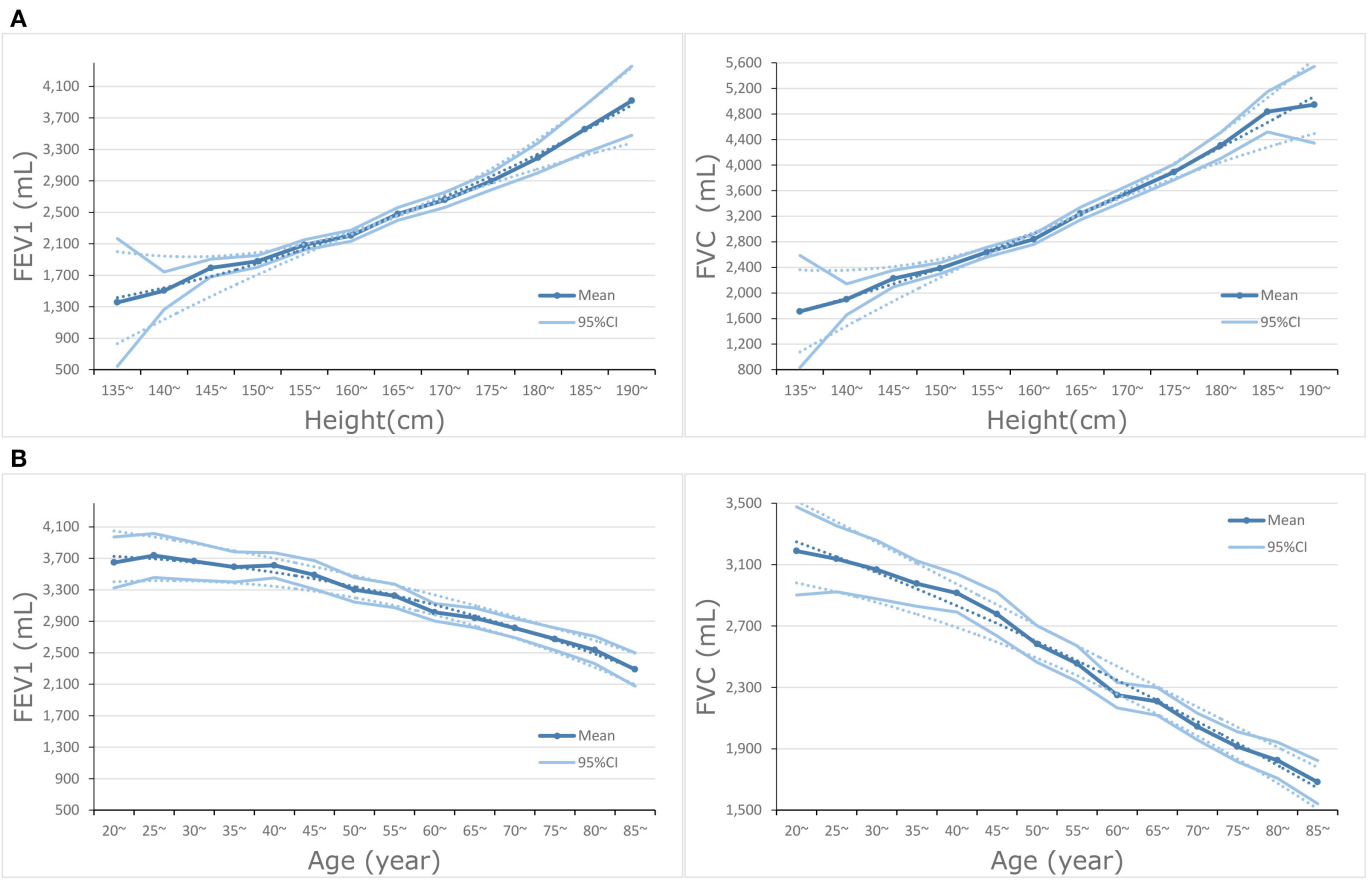
**(A)** Dose-response association between height and FEV1 and FVC. **(B)** Dose-response association between age and FEV1 and FVC.

In the subgroup analysis of hypertension, lower FEV1 was associated with a higher risk of all-cause mortality in with and without hypertension subgroups. The associations of CHD and CVD were only observed in the hypertension subgroup; HRs were 3.87 (95%CI: 1.21–12.40) and 4.30 (95%CI: 1.57–11.79), respectively. The associations of FVC were significant in subgroups with and without hypertension. Stratified by the history of dyslipidemia, the association of FEV1 and CHD became significant in both subgroups. The associations of FEV1 and FVC with CHD, CVD, and all-cause mortality were observed in the dyslipidemia subgroup (all *P* for trend <0.050), but not associated in the without dyslipidemia subgroup. A total of 936 participants died within 2 years of follow-up and 951 participants had a medical history of diseases related to lung function and excluding them resulted in no substantial changes in the associations of FEV1 and FVC with CVD and all-cause mortality (Supplementary Tables 1.1,1.2). FEV1 and FVC increased with height, but the opposite trend appeared with age (Figures 2A,B).

## Discussion

In this prospective cohort study, lower FEV1 and FVC were associated with a higher risk of CHD, CVD, and all-cause mortality among USA adults with diabetes. After adjustment for cardiovascular risk factors and other covariates related to lung function, the HRs were not attenuated. In addition, we also observed the association between lower FVC and cancer mortality, whereas lower FEV1 was not associated with cancer mortality after adjusting the confounders.

In previous studies, decreased lung function was associated with a higher cardiovascular risk among the general population (2, 5, 7, 25, 26). A cohort study on the general Chinese people found that each 5% decrease in FEV1/FVC was associated with a 0.47% increase in 10-year CVD risk (*P* < 0.001) (25). Similarly, in a population-based prospective cohort study with a follow-up of over 18 years, there is a reported decrease of 28%-35% mortality risk from CVD for every 70 L/s increase in FEV1 (26). In a cohort study of 14,503 adults from the Moli-sani study, the HRs of FEV1% pred and FVC% pred in the lowest quartile for CVD mortality were 1.59 (95%CI: 1.02–2.47) and 1.97 (95%CI: 1.97–3.08), respectively (2). In the same population (NHANS III) of the present study, the reduced lung function was associated with CVD mortality (HR of FEV1% pred: 1.7, 95%CI: 1.4–2.1; HR of FVC% pred: 2.1, 95%CI: 1.7–2.6) among the general population (5). The present study reinforced the association of lung function with CVD mortality. Moreover, previous studies reported an inverse association of reduced lung function with CHD (4, 5, 8, 26, 27). A prospective study of 4,434 men with no history of CVD (CHD or stroke) and diabetes demonstrated that lower FEV1 levels were associated with a higher risk of fatal CHD (Relative risk: 1.63; 95%CI: 1.03–2.67), but not associated with higher risks of major CHD and non-fatal MI (8). In a prospective study with a 29-year follow-up of the Buffalo Health Study cohort, the authors found that lung function was inversely associated with the risk of CHD (27). The HRs of FEV1% pred in the 1st quintile for CHD mortality were 2.11 (95%CI: 1.20–3.71) among men and 1.96 (95%CI:0.99–3.88) among women. In a cohort study of 15,411 adults, a lower relative FEV1 was shown to be associated with a higher risk of CHD (HRs: 1.56 (1.26–1.92) for men and 1.88 (1.44–2.47) for women) (4). The previous study investigating the general population of the same cohort as the present study reported a similar risk estimate, an approximately 2-fold increased risk of CHD mortality, compared with other previous studies (5).

Our findings indicated a potentially stronger association of lung function with CVD or CHD mortality in the population with diabetes than that in the general population. They were in line with some previous studies. In a cohort study of 1,743 adults, compared with clinically normal participants (as reference), the adjusted odds ratio of participants with diabetes depicted a stronger association between FEV1 (HR: 1.67; *P* < 0.01) and FVC (HR:1.51; *P* = 0.02) with overall mortality, which was nearly a 1.5-fold risk with comparison to the general population (1). In addition, logistic regression analysis elaborated that the adjusted risk of CVD mortality was reinforced for all participants with any lung function impairment, current or former smoker, and patients with type 2 diabetes (1), which was in accordance with our findings. Hedblad et al. examined the association of FVC with insulin resistance and CVD incidence and found that subjects who had developed insulin resistance had the highest risk of CVD events (28). Taking the high FVC group without insulin resistance as a reference, the adjusted relative risk of low FVC among subjects with insulin resistance was 1.7 (95%CI: 1.02–2.70).

Additionally, after excluding those who had developed diabetes at the follow-up examination, the result did not change substantially. However, in some studies, whether the association is more robust in a population with diabetes is inconclusive. For instance, Wannamethe et al. found that exclusion of men with diabetes resulted in little difference in the association of lung function with fatal or non-fatal CHD events (8).

Although lung function has been extensively recognized and considered as an effective predictor of CVD, the mechanism underlying the association still requires evidence. Smoking has been considered responsible for the association ever (29). However, the association was additionally observed in non-smokers (4, 9, 30). Furthermore, in a study examining the extent to which risk factors explain the association, smoking history was only reduced by 4.9%, suggesting that smoking history was not the predominant explanation for this association (10). Some other potential mechanisms have been nominated. For example, few studies proposed that poor lung function may result from long-term exposure to air pollution or diesel exhaust fumes, finally causing diseases or death (27). Recently, inflammation has seized considerable attention regarding the association between lung function and CVD mortality. Sabia et al. highlighted the prominence of inflammatory markers, which account for the association of lung function with mortality more than any other risk factors (10). C-reactive protein (CRP) has been consistently regarded as a reliable indicator of underlying low-grade systemic inflammation and as a critical biomarker for the onset and mortality of CVD (31–34). There is an inverse association between FVC and CRP in a cross-sectional study (35). Association has also been identified between FVC and plasma levels of inflammation-sensitive plasma proteins, another inflammatory marker (3). Engström et al. also suggested that this association contributes to the risk of CVD mortality (relative risk: 3.7; 95%CI: 2.2–6.3) in men with low FVC levels. Notably, previous studies reported that the subjects with elevated CRP had an approximately 2-fold increased risk of cardiac injury (5, 6).

In the study including all subjects of NHANES III, high CRP significantly increased HR of CVD mortality among subjects with the lowest FVC% pred or FEV1% pred (5). Our findings are consistent with their results (data not shown), implying that systemic inflammation greatly affects the connection between declined lung function and CVD mortality. Besides, low-grade systemic inflammation was associated with diabetes (13, 36), and previous studies indicated that CRP was an independent predictor of cardiovascular risk in the population with diabetes (37–40). Since the association of lung function with CVD mortality lies in systemic inflammation, especially CRP, and CRP could convey independent prediction information of cardiovascular risk in subjects with diabetes, a stronger association of lung function with CVD mortality in subjects with diabetes may be established. In a previous study, poor lung function was associated with the increased risk of fatal events and case fatality of CHD (HR: 1.63; 95% CI: 1.03–2.67), and inflammatory pathway adjustment further attenuated FEV1 with both diabetes and the association of both diabetes and fatal CHD, suggesting that reduced lung function may be a potential factor linking diabetes to increased risk of CHD and increased susceptibility to a fatal episode in the event of a cardiac event (8). However, few studies suggested that the magnitude of association between CRP and CVD mortality is comparable in people with and without diabetes (39, 41). Therefore, further studies will need to provide the magnitude of this association in people with and without diabetes.

Notably, in the present study, the association of FEV1 with CVD and CHD mortality was only observed in the subgroup, in which subjects with over 25 BMI were included. BMI is a known and well-established risk factor for both CVD, CHD, and diabetes. A previous study reported a similar result (42). Each 1 kg/m^2^ increase in BMI resulted in a 5% increase in the risk of CVD mortality for a reduction in relative FEV1 of 10% (42). Nevertheless, few studies suggested no substantial difference in HRs in the subgroups stratified by BMI (5, 7, 26). The inconclusive results between previous and present studies may be attributed to an averagely greater BMI in people with diabetes than that in the general population. Similar findings were observed in the subgroups stratified by medical history of dyslipidemia. The associations of both FVC and FEV1 with CVD mortality were only identified in the subjects with dyslipidemia. Dyslipidemia, as an important cardiovascular risk factor, was related to decreased FVC% pred and FEV1% pred (43). The diagnosis criterion is usually the reduced high-density lipoprotein (HDL), which is <40 mg/dl. A previous study indicated the association of low HDL with reduced lung function, owing to its roles in reverse cholesterol transport and anti-inflammation (44). Therefore, dyslipidemia may affect the association of lung function with CVD mortality. Further studies, however, are required to identify the effect modification of dyslipidemia. In addition, we only observed a significant association of FEV1 with CVD and CHD in the stratum with a history of hypertension. The result is consistent with the previous study. Taking clinically normal participants as a reference, the ORs of FEV1 and FVC with risk of CVD in participants with hypertension were 2.15 (1.63–2.83) and 2.19 (1.66–2.88), respectively (1). For CHD, it was shown that hypertension was associated with insulin resistance and glucose intolerance, with evidence of endothelial dysfunction, which is mainly responsible for the increased risk of CHD mortality (45). In our stratified analysis, the results of FEV1 and FVC are not consistent. For instance, there was a significant association of FEV1 with CHD mortality in the stratum with over 25 BMI but not in another subgroup, while we observed significant associations of FVC in both subgroups. This may be attributed to the different prediction effects of these two spirometric parameters. Previous studies also reported that FEV 1 was a stronger predictor for CVD mortality in a population with chronic obstructive pulmonary disease than FVC (46) or FVC was superior to FEV1 in the general population (47, 48). However, the evidence was not conclusive.

To our knowledge, the present study is the first to investigate the association of lung function with CVD mortality in people with diabetes and hypothesize a stronger association in people with diabetes than in the general population. FEV1 and FVC are spirometric parameters extensively used in lung function tests, which can be a non-invasive approach to provide additional prognostic and predictive information on CVD and the risk of further cardiovascular events. Our findings imply that FEV1 and FVC can be utilized in spirometric tests and for the prevention and management of CVD, especially for those with diabetes or metabolic syndrome. Meanwhile, exploring emerging data for the association in different population can provide better health outcomes for patients and sufficient evidence for the management of the chronic disease.

The present study has some limitations. First, the spirometric measurement in the present study may be inadequate, owing to only one testing at the baseline. Extreme or inaccurate values by the single lung function test may affect the accuracy of results. Second, the period from the baseline to the follow-up outcome was excessively long. We recognize that lung function was not unchangeable, and the influence of lung function changes on the risk of CVD mortality was not addressed. Third, CVD mortality may be overestimated, especially for the elderly (49), because the NHANES III Linked Mortality File overly attributed the cause of death to CVD mortality. Lastly, we cannot determine all confounding effects although we have already adjusted for some known cardiovascular risk factors and lung function-related covariates.

## Conclusion

Declined lung function was associated with a higher risk of CVD and all-cause mortality in people with diabetes. Lung function tests, especially FEV1 and FVC, should be encouraged to provide prognostic and predictive information for the management of CVD and all-cause mortality in patients with diabetes.

## Data availability statement

The original contributions presented in the study are included in the article/Supplementary material, further inquiries can be directed to the corresponding author/s.

## Author contributions

JC supervised the study. JC, NH, and CT designed the study. NH, SL, WM, XZ, KL, and HS collected and organized the data. NH analyzed the data. JC, NH, CT, and SL interpreted the results. CT wrote the first draft. All authors read and approved the final manuscript.

## Funding

This work was supported by the National Natural Science Foundation of China (grant number 82173626), the Fundamental Research Funds for the Central Universities (grant number 2020YJ066), the Fundamental Research Funds for the Health Commission of Hubei Province (grant number SWSZFY2021), and the Fundamental Research Funds for the Health Commission of Wuhan (grant number WHWSZFY2021).

## Conflict of interest

The authors declare that the research was conducted in the absence of any commercial or financial relationships that could be construed as a potential conflict of interest.

## Publisher's note

All claims expressed in this article are solely those of the authors and do not necessarily represent those of their affiliated organizations, or those of the publisher, the editors and the reviewers. Any product that may be evaluated in this article, or claim that may be made by its manufacturer, is not guaranteed or endorsed by the publisher.
