# Supplementary material for: Association of lung function with the risk of cardiovascular diseases and all-cause mortality in patients with diabetes: Results from NHANES III 1988-1994
Source: Front Cardiovasc Med. 2022 Sep 9;9:976817. doi: 10.3389/fcvm.2022.976817 (PMC9500166; doi:10.3389/fcvm.2022.976817)
Supplement: Supplementary file 1 [file Data_Sheet_1.docx]

**Supplementary Table 1.1** Associations of FEV1 and FVC With Coronary heart disease, Cardiovascular, Cancer and All-Cause Mortality in U.S. Adults Aged at Least 20 Years, Excluding Deaths During the First 2 Years of Follow-up.

| 1615 | FEV1 | | | | |  | FVC | | | | |  |
| --- | --- | --- | --- | --- | --- | --- | --- | --- | --- | --- | --- | --- |
|  | Q1  ≥2883 | Q2  2307.5-2882.9 | Q3  1818-2307.4 | Q4  <1818 | *P for trend* |  | Q1  ≥3750.5 | Q2  2995.5-3750.4 | Q3  2379-2995.4 | Q4  <2379 | *P for trend* | |
|  |  |  |  |  |  |  |  |  |  |  |  | |
| **CHD mortality** |  |  |  |  |  |  |  |  |  |  |  | |
| Deaths, No. (%) | 50(11.0) | 65(13.9) | 87(26.3) | 83(20.5) | <0.001 |  | 54(12.2) | 68(15.4) | 74(21.6) | 89(21.6) | 0.002 | |
| Deaths/person-years | 685/7796 | 829/7082 | 828/5936 | 811/4244 |  |  | 701/7399 | 781/6812 | 851/6372 | 820/4475 |  | |
| Unadjusted | 1.00 [Reference] | 1.32(0.63,2.75) | 3.37(1.94,5.85) | 3.54(1.83,6.86) | <0.001 |  | 1.00 [Reference] | 1.32(0.71,2.46) | 2.12(1.23,3.64) | 3.04(1.81,5.11) | <0.001 | |
| Model 1 | 1.00 [Reference] | 1.25(0.63,2.47) | 2.80(1.51,5.18) | 2.44(1.06,5.59) | 0.015 |  | 1.00 [Reference] | 1.48(0.84,2.62) | 2.22(1.16,4.25) | 2.70(1.18,6.16) | 0.016 | |
| Model 2 | 1.00 [Reference] | 1.23(0.62,2.43) | 2.65(1.47,4.80) | 2.25(0.98,5.21) | 0.025 |  | 1.00 [Reference] | 1.58(0.93,2.71) | 2.19(1.19,4.04) | 2.69(1.15,6.26) | 0.020 | |
| Model 3 | 1.00 [Reference] | 1.23(0.56,2.70) | 2.65(1.31,5.37) | 2.48(0.84,7.29) | 0.031 |  | 1.00 [Reference] | 1.90(1.09,3.33) | 3.05(1.55,5.98) | 4.79(1.87,12.25) | 0.001 | |
| Model 4 | 1.00 [Reference] | 1.25(0.57,2.74) | 2.74(1.37,5.45) | 2.70(0.97,7.58) | 0.016 |  | 1.00 [Reference] | 2.10(1.27,3.48) | 3.32(1.78,6.19) | 6.03(2.85,12.74) | <0.001 | |
|  |  |  |  |  |  |  |  |  |  |  |  | |
| **CVD mortality** |  |  |  |  |  |  |  |  |  |  |  | |
| Deaths, No. (%) | 63(13.4) | 77(15.5) | 112(32.7) | 106(24.4) | <0.001 |  | 68(14.5) | 79(17.3) | 96(26.7) | 115(26.8) | <0.001 | |
| Deaths/person-years | 858/7796 | 967/7082 | 1153/5936 | 1000/4244 |  |  | 878/7399 | 919/6812 | 1132/6372 | 1048/4475 |  | |
| Unadjusted | 1.00 [Reference] | 1.20(0.63,2.29) | 3.44(2.07,5.70) | 3.48(2.02,5.99) | <0.001 |  | 1.00 [Reference] | 1.25(0.74,2.13) | 2.21(1.35,3.63) | 3.18(2.04,4.94) | <0.001 | |
| Model 1 | 1.00 [Reference] | 1.08(0.59,1.98) | 2.58(1.48,4.49) | 2.16(1.07,4.36) | 0.011 |  | 1.00 [Reference] | 1.34(0.81,2.22) | 2.11(1.15,3.86) | 2.56(1.26,5.21) | 0.008 | |
| Model 2 | 1.00 [Reference] | 1.07(0.59,1.95) | 2.47(1.46,4.18) | 2.05(1.01,4.17) | 0.017 |  | 1.00 [Reference] | 1.43(0.89,2.30) | 2.09(1.21,3.61) | 2.62(1.27,5.38) | 0.008 | |
| Model 3 | 1.00 [Reference] | 1.10(0.56,2.19) | 2.59(1.41,4.76) | 2.41(0.98,5.94) | 0.012 |  | 1.00 [Reference] | 1.83(1.10,3.02) | 3.15(1.70,5.84) | 5.23(2.28,12.03) | <0.001 | |
| Model 4 | 1.00 [Reference] | 1.14(0.58,2.27) | 2.76(1.52,5.03) | 2.57(1.09,6.10) | 0.005 |  | 1.00 [Reference] | 1.99(1.26,3.14) | 3.39(1.93,5.96) | 6.33(3.23,12.4) | <0.001 | |
|  |  |  |  |  |  |  |  |  |  |  |  | |
| **Cancer mortality** |  |  |  |  |  |  |  |  |  |  |  | |
| Deaths, No. (%) | 34(7) | 49(10.9) | 54(14.3) | 42(11.1) | 0.121 |  | 41(10) | 54(10.7) | 45(10.4) | 39(10.6) | 0.439 | |
| Deaths/person-years | 383/7796 | 576/7082 | 563/5936 | 415/4244 |  |  | 424/7399 | 619/6812 | 519/6372 | 375/4475 |  | |
| Unadjusted | 1.00 [Reference] | 1.60(0.80,3.23) | 2.78(1.55,50) | 2.87(1.42,5.80) | <0.001 |  | 1.00 [Reference] | 1.11(0.53,2.35) | 1.23(0.58,2.61) | 1.79(0.95,3.36) | 0.162 | |
| Model 1 | 1.00 [Reference] | 1.49(0.68,3.25) | 2.28(0.92,5.69) | 1.93(0.67,5.58) | 0.153 |  | 1.00 [Reference] | 1.49(0.68,3.25) | 2.28(0.92,5.69) | 1.93(0.67,5.58) | 0.615 | |
| Model 2 | 1.00 [Reference] | 1.47(0.68,3.18) | 2.03(0.77,5.37) | 1.82(0.59,5.66) | 0.237 |  | 1.00 [Reference] | 1.24(0.57,2.70) | 0.99(0.38,2.65) | 1.38(0.50,3.76) | 0.619 | |
| Model 3 | 1.00 [Reference] | 1.52(0.69,3.36) | 2.17(0.68,6.93) | 2.12(0.47,9.61) | 0.235 |  | 1.00 [Reference] | 1.68(0.69,4.04) | 1.57(0.45,5.48) | 2.83(0.74,10.89) | 0.178 | |
| Model 4 | 1.00 [Reference] | 1.44(0.66,3.15) | 2.09(0.70,6.24) | 2.26(0.55,9.28) | 0.202 |  | 1.00 [Reference] | 1.77(0.69,4.54) | 1.68(0.46,6.18) | 3.40(0.87,13.30) | 0.117 | |
|  |  |  |  |  |  |  |  |  |  |  |  | |
| **All-cause mortality** |  |  |  |  |  |  |  |  |  |  |  | |
| Deaths, No. (%) | 217(48.1) | 262(55.2) | 306(75.7) | 328(88.3) | <0.001 |  | 243(53.4) | 262(56.4) | 284(71.4) | 324(82.7) | <0.001 | |
| Deaths/person-years | 3001/7796 | 3425/7082 | 3574/5936 | 3254/4244 |  |  | 3259/7399 | 3234/6812 | 3619/6372 | 3141/4475 |  | |
| Unadjusted | 1.00 [Reference] | 1.19(0.88,1.63) | 2.28(1.70,3.05) | 3.71(2.75,5.00) | <0.001 |  | 1.00 [Reference] | 1.10(0.83,1.46) | 1.63(1.18,2.25) | 2.80(2.00,3.90) | <0.001 | |
| Model 1 | 1.00 [Reference] | 1.15(0.89,1.49) | 1.92(1.35,2.73) | 2.62(1.67,4.11) | <0.001 |  | 1.00 [Reference] | 1.22(0.94,1.58) | 1.65(1.12,2.43) | 2.43(1.45,4.05) | 0.001 | |
| Model 2 | 1.00 [Reference] | 1.13(0.88,1.43) | 1.76(1.31,2.37) | 2.41(1.57,3.71) | <0.001 |  | 1.00 [Reference] | 1.28(0.99,1.67) | 1.56(1.12,2.19) | 2.43(1.43,4.14) | 0.002 | |
| Model 3 | 1.00 [Reference] | 1.19(0.88,1.60) | 1.93(1.39,2.67) | 2.96(1.84,4.79) | <0.001 |  | 1.00 [Reference] | 1.48(1.10,1.99) | 1.99(1.35,2.95) | 3.67(1.99,6.78) | <0.001 | |
| Model 4 | 1.00 [Reference] | 1.16(0.87,1.55) | 1.85(1.36,2.51) | 2.96(1.92,4.55) | <0.001 |  | 1.00 [Reference] | 1.51(1.12,2.04) | 2.01(1.38,2.92) | 3.98(2.37,6.68) | <0.001 | |

Values are n or weighted hazard ratio (95% confidence interval).

Model 1: adjusted for sex, age.

Model 2: model 1 + education, BMI, alcohol, and smoking.

Model 3: model 2 + HDL-cholesterol, serum C-reactive protein, serum albumin, fev1/fvc and fev1%pred or fvc% pred.

Model 4: model 3 + history of hypertension, history of dyslipidemia, history of whistling and/or wheezing, persist phlegm, persist cough, asthma, history of chronic bronchitis, history of emphysema and history of cold or flu.

CHD = coronary heart disease.

CVD = cardiovascular disease.

BMI = body mass index.

FEV1 = forced expiratory volume in 1 s.

FVC = forced vital capacity.

**Supplementary Table 1.2** Associations of FEV1 and FVC With Coronary heart disease, Cardiovascular, Cancer and All-Cause Mortality in U.S. Adults Aged at Least 20 Years, Excluding Participants with Histories of Disease (Asthma, Chronic bronchitis, Emphysema)

|  | FEV1 | | | | |  | FVC | | | | |
| --- | --- | --- | --- | --- | --- | --- | --- | --- | --- | --- | --- |
|  | Q1  ≥2883 | Q2  2307.5-2882.9 | Q3  1818-2307.4 | Q4  <1818 | *P for trend* |  | Q1  ≥3750.5 | Q2  2995.5-3750.4 | Q3  2379-2995.4 | Q4  <2379 | *P for trend* |
| N=1487 |  |  |  |  |  |  |  |  |  |  |  |
| **CHD mortality** |  |  |  |  |  |  |  |  |  |  |  |
| Deaths, No. (%) | 52(13.0) | 60(14.1) | 80(27.1) | 78(21.5) | <0.001 |  | 56(13.9) | 64(17.0) | 68(20.5) | 82(22.8) | 0.010 |
| Deaths/person-years | 641/7125 | 718/6170 | 715/5144 | 616/3262 |  |  | 658/6694 | 662/5874 | 736/5526 | 634/3607 |  |
| Unadjusted | 1.00 [Reference] | 1.12(0.58,2.19) | 2.82(1.72,4.62) | 2.95(1.82,4.78) | <0.001 |  | 1.00 [Reference] | 1.28(0.67,2.43) | 1.69(0.97,2.94) | 2.70(1.64,4.44) | <0.001 |
| Model 1 | 1.00 [Reference] | 1.12(0.59,2.14) | 2.52(1.38,4.62) | 2.30(1.07,4.93) | 0.015 |  | 1.00 [Reference] | 1.56(0.81,2.98) | 2.04(0.99,4.19) | 2.69(1.14,6.35) | 0.024 |
| Model 2 | 1.00 [Reference] | 1.09(0.58,2.05) | 2.37(1.35,4.19) | 2.06(0.99,4.28) | 0.024 |  | 1.00 [Reference] | 1.65(0.84,3.24) | 2.01(0.99,4.08) | 2.65(1.09,6.40) | 0.030 |
| Model 3 | 1.00 [Reference] | 1.18(0.56,2.46) | 2.70(1.48,4.93) | 2.73(1.05,7.11) | 0.010 |  | 1.00 [Reference] | 2.01(1.06,3.84) | 3.10(1.64,5.85) | 5.23(2.29,11.94) | <0.001 |
| Model 4 | 1.00 [Reference] | 1.18(0.57,2.42) | 2.52(1.36,4.65) | 2.75(1.11,6.82) | 0.009 |  | 1.00 [Reference] | 2.07(1.13,3.79) | 2.97(1.55,5.68) | 5.49(2.61,11.58) | <0.001 |
|  |  |  |  |  |  |  |  |  |  |  |  |
| **CVD mortality** |  |  |  |  |  |  |  |  |  |  |  |
| Deaths, No. (%) | 65(15.6) | 74(17.1) | 101(33.0) | 99(25.1) | <0.001 |  | 72(16.4) | 75(20.3) | 87(25.4) | 105(27.1) | 0.0007 |
| Deaths/person-years | 801/7125 | 841/6170 | 975/5144 | 744/3262 |  |  | 837/6694 | 752/5874 | 956/5526 | 816/3607 |  |
| Unadjusted | 1.00 [Reference] | 1.13(0.62,2.05) | 2.83(1.79,4.49) | 2.81(1.83,4.32) | <0.001 |  | 1.00 [Reference] | 1.29(0.74,2.26) | 1.76(1.05,2.96) | 2.68(1.73,4.16) | <0.001 |
| Model 1 | 1.00 [Reference] | 1.07(0.58,1.96) | 2.29(1.35,3.91) | 1.98(1.02,3.84) | 0.015 |  | 1.00 [Reference] | 1.51(0.83,2.74) | 1.96(1.01,3.82) | 2.46(1.16,5.18) | 0.018 |
| Model 2 | 1.00 [Reference] | 1.05(0.59,1.89) | 2.26(1.38,3.70) | 1.92(0.98,3.76) | 0.020 |  | 1.00 [Reference] | 1.66(0.86,3.20) | 2.07(1.09,3.96) | 2.67(1.17,6.07) | 0.018 |
| Model 3 | 1.00 [Reference] | 1.16(0.61,2.22) | 2.68(1.59,4.51) | 2.64(1.15,6.04) | 0.003 |  | 1.00 [Reference] | 2.12(1.13,3.99) | 3.34(1.83,6.11) | 5.62(2.51,12.6) | <0.001 |
| Model 4 | 1.00 [Reference] | 1.17(0.64,2.15) | 2.57(1.56,4.24) | 2.74(1.28,5.86) | 0.001 |  | 1.00 [Reference] | 2.23(1.21,4.09) | 3.34(1.80,6.17) | 6.20(2.89,13.31) | <0.001 |
|  |  |  |  |  |  |  |  |  |  |  |  |
| **Cancer mortality** |  |  |  |  |  |  |  |  |  |  |  |
| Deaths, No. (%) | 33(7.3) | 42(9.8) | 51(13.0) | 37(9.4) | 0.124 |  | 36(9.5) | 45(9.8) | 46(8.9) | 36(9.7) | 0.391 |
| Deaths/person-years | 373/7295 | 479/6247 | 492/5193 | 309/3402 |  |  | 372/6855 | 514/5942 | 472/5610 | 297/3731 |  |
| Unadjusted | 1.00 [Reference] | 1.39(0.70,2.77) | 2.37(1.19,4.71) | 2.25(1.13,4.49) | 0.006 |  | 1.00 [Reference] | 1.07(0.48,2.40) | 1.07(0.50,2.29) | 1.68(0.83,3.41) | 0.288 |
| Model 1 | 1.00 [Reference] | 1.18(0.58,2.41) | 1.66(0.65,4.24) | 1.33(0.50,3.53) | 0.444 |  | 1.00 [Reference] | 1.08(0.49,2.41) | 0.94(0.38,2.34) | 1.16(0.47,2.90) | 0.809 |
| Model 2 | 1.00 [Reference] | 1.16(0.55,2.43) | 1.50(0.56,4.04) | 1.21(0.41,3.57) | 0.622 |  | 1.00 [Reference] | 1.16(0.51,2.63) | 0.87(0.35,2.12) | 1.15(0.43,3.09) | 0.873 |
| Model 3 | 1.00 [Reference] | 1.13(0.49,2.58) | 1.44(0.47,4.39) | 1.17(0.27,4.98) | 0.677 |  | 1.00 [Reference] | 1.44(0.59,3.49) | 1.25(0.40,3.90) | 2.04(0.52,7.96) | 0.362 |
| Model 4 | 1.00 [Reference] | 1.14(0.50,2.59) | 1.42(0.49,4.16) | 1.24(0.31,4.99) | 0.640 |  | 1.00 [Reference] | 1.45(0.58,3.65) | 1.27(0.41,3.99) | 2.27(0.59,8.75) | 0.306 |
|  |  |  |  |  |  |  |  |  |  |  |  |
| **All-cause mortality** |  |  |  |  |  |  |  |  |  |  |  |
| Deaths, No. (%) | 216(50.5) | 244(57.5) | 287(75.0) | 294(87.5) | <0.001 |  | 238(55.6) | 237(57.6) | 267(69.3) | 299(82.6) | <0.001 |
| Deaths/person-years | 2775/7125 | 3032/6170 | 3085/5144 | 2532/3262 |  |  | 2978/6694 | 2761/5874 | 3125/5526 | 2559/3607 |  |
| Unadjusted | 1.00 [Reference] | 1.18(0.87,1.62) | 2.08(1.53,2.83) | 3.32(2.45,4.49) | <0.001 |  | 1.00 [Reference] | 1.08(0.80,1.46) | 1.44(1.03,2.03) | 2.59(1.84,3.65) | <0.001 |
| Model 1 | 1.00 [Reference] | 1.15(0.85,1.54) | 1.73(1.15,2.61) | 2.40(1.43,4.03) | 0.001 |  | 1.00 [Reference] | 1.24(0.92,1.66) | 1.53(0.99,2.36) | 2.29(1.33,3.95) | 0.004 |
| Model 2 | 1.00 [Reference] | 1.11(0.83,1.48) | 1.60(1.14,2.23) | 2.16(1.31,3.54) | 0.002 |  | 1.00 [Reference] | 1.30(0.97,1.74) | 1.46(1.01,2.11) | 2.26(1.29,3.97) | 0.006 |
| Model 3 | 1.00 [Reference] | 1.24(0.87,1.75) | 1.91(1.35,2.70) | 2.99(1.77,5.02) | <0.001 |  | 1.00 [Reference] | 1.53(1.12,2.08) | 2.03(1.40,2.94) | 3.72(2.06,6.70) | <0.001 |
| Model 4 | 1.00 [Reference] | 1.23(0.88,1.71) | 1.83(1.31,2.55) | 2.94(1.83,4.70) | <0.001 |  | 1.00 [Reference] | 1.52(1.13,2.07) | 1.96(1.37,2.81) | 3.79(2.26,6.35) | <0.001 |

Values are n or weighted hazard ratio (95% confidence interval).

Model 1: adjusted for sex, age.

Model 2: model 1 + education, BMI, alcohol, and smoking.

Model 3: model 2 + HDL-cholesterol, serum C-reactive protein, serum albumin, fev1/fvc and fev1%pred or fvc% pred.

Model 4: model 3 + history of hypertension, history of dyslipidemia, history of whistling and/or wheezing, persist phlegm, persist cough, and history of cold or flu.

CHD = coronary heart disease.

CVD = cardiovascular disease.

BMI = body mass index.

FEV1 = forced expiratory volume in 1 s.

FVC = forced vital capacity.
